# Supplementary material for: Novel Driver Strength Index highlights important cancer genes in TCGA PanCanAtlas patients
Source: PeerJ. 2022 Aug 11;10:e13860. doi: 10.7717/peerj.13860 (PMC9375969; doi:10.7717/peerj.13860)
Supplement: Supplemental Information 2 [file peerj-10-13860-s002.zip › COHORTS/genes plots/detailed/2021_8_16_14_9_distribution_events_detailed_5.pdf]

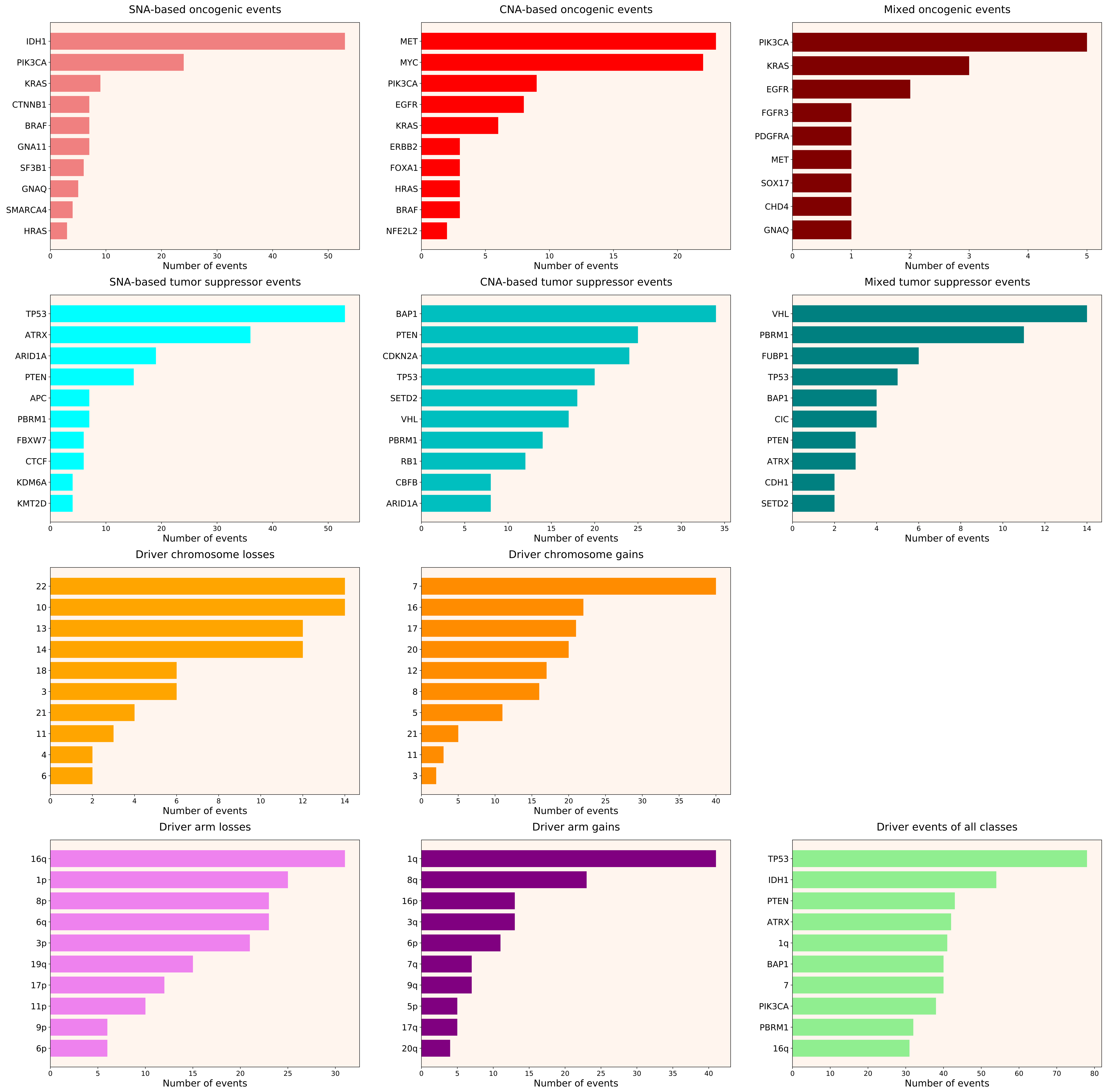
SNA-based oncogenic events
IDH1
PIK3CA
KRAS
CTNNB1
BRAF
GNA11
SF3B1
GNAQ
SMARCA4
HRAS
Number of events
CNA-based oncogenic events
MET
MYC
PIK3CA
EGFR
KRAS
ERBB2
FOXA1
HRAS
BRAF
NFE2L2
Number of events
Mixed oncogenic events
PIK3CA
KRAS
EGFR
FGFR3
PDGFRA
MET
SOX17
CHD4
GNAQ
Number of events
SNA-based tumor suppressor events
TP53
ATRX
ARID1A
PTEN
APC
PBRM1
FBXW7
CTCF
KDM6A
KMT2D
Number of events
CNA-based tumor suppressor events
BAP1
PTEN
CDKN2A
TP53
SETD2
VHL
PBRM1
RB1
CBFB
ARID1A
Number of events
Mixed tumor suppressor events
VHL
PBRM1
FUBP1
TP53
BAP1
CIC
PTEN
ATRX
CDH1
SETD2
Number of events
Driver chromosome losses
22
10
13
14
18
3
21
11
4
6
Number of events
Driver chromosome gains
7
16
17
20
12
8
5
21
11
3
Number of events
Driver arm losses
16q
1p
8p
6q
3p
19q
17p
11p
9p
6p
Number of events
Driver arm gains
1q
8q
16p
3q
6p
7q
9q
5p
17q
20q
Number of events
Driver events of all classes
TP53
IDH1
PTEN
ATRX
1q
BAP1
7
PIK3CA
PBRM1
16q
Number of events
